# Supplementary material for: Awareness of Locomotive Syndrome and Factors Associated with Awareness: A Community-Based Cross-Sectional Study
Source: Int J Environ Res Public Health. 2020 Oct 5;17(19):7272. doi: 10.3390/ijerph17197272 (PMC7579478; doi:10.3390/ijerph17197272)
Supplement: Supplementary file 1 [file ijerph-17-07272-s001.pdf]

**Supplementary**

**Table S1.** Comparison of age and sex of study participants and non-participants.

| | | Participants | | Non-Participants | | Chi-square test |
|---|---|---|---|---|---|---|
| | | n | % | n | % | |
| Age group | 40-49 | 213 | 11.8 | 285 | 20.7 | |
| | 50-59 | 334 | 18.5 | 270 | 19.6 | |
| | 60-69 | 521 | 28.9 | 270 | 19.6 | |
| | 70-79 | 413 | 22.9 | 209 | 15.2 | |
| | 80 or over | 323 | 17.9 | 343 | 24.9 | <0.001 |
| Sex | Female | 1005 | 55.7 | 674 | 49.0 | |
| | Male | 799 | 44.3 | 703 | 51.0 | <0.001 |
